# Enhancement of vindoline and catharanthine production in *Catharanthus roseus* by LED light and plasma activated water

**DOI:** 10.1371/journal.pone.0315542

**Published:** 2024-12-31

**Authors:** Alessandro Quadri, Alberto Barbaresi, Patrizia Tassinari, Assunta Bertaccini, Nicoletta Contaldo, Laura Mercolini, Michele Protti, Roberto Montalbetti, Romolo Laurita, Daniele Torreggiani

**Affiliations:** 1 Department of Agricultural and Food Sciences (DISTAL), University of Bologna, Bologna, Italy; 2 Institute for Sustainable Plant Protection (IPSP), Bari—National Research Council, Bari, Italy; 3 Department of Pharmacy and Biotechnology (FABIT), University of Bologna, Bologna, Italy; 4 Department of Industrial Engineering (DIN), University of Bologna, Bologna, Italy; Kenyatta University School of Pure and Applied Sciences, KENYA

## Abstract

This study aimed to increase the concentrations of vindoline (VDL) and catharanthine (CAT) in *Catharanthus roseus* plants cultivated in an indoor farming system using artificial lighting and plasma-activated water (PAW). After a 61-days pre-treatment period under fluorescent lamps, plants were exposed to four treatments: white light (W) from the same fluorescent lamps, red light (R) from LEDs, W with PAW, and R with PAW. These combinations were evaluated at two sampling times: 45 days (T1) and 70 days (T2) after the end of pre-treatment (DAP). Results showed that R combined with PAW significantly increased VDL and CAT concentrations compared to other combinations. In particular, with PAW, R produced significantly higher VDL and CAT concentrations than W, while without PAW, VDL and CAT concentrations were comparable under W and R. Regardless of the light conditions, VDL and CAT concentrations were higher with PAW. Moreover, VDL and CAT concentrations increased from T1 to T2, reaching higher levels under R or PAW at T2. At the same sampling time, VDL and CAT levels were generally higher in plants exposed to R and in those treated with PAW. The highest VDL and CAT concentrations were observed with combined R and PAW at T2. The study concluded that: (1) VDL and CAT concentrations increase with plant age; (2) PAW enhances VDL and CAT concentrations, with its effect becoming more pronounced from T1 to T2; (3) R contributes to VDL and CAT biosynthesis, but its impact becomes significant only when combined with PAW and its effect is amplified from T1 to T2; (4) regardless of the sampling time, the treatment with R and PAW maximizes the VDL and CAT concentrations; (5) R combined with PAW at T2 is the most effective treatment; (6) if harvest timing cannot be delayed, using R and PAW offers substantial benefits.

## 1. Introduction

*Catharanthus roseus* (L.) G. Don., commonly known as periwinkle, is a medicinal plant of great pharmaceutical interest due to its production of dimeric indole alkaloids (DIAs), including vinblastine (VBL) and vincristine (VCR). These alkaloids are widely used as chemotherapeutic agents [1]. VBL is employed in the treatment of various cancers, including Hodgkin’s disease, testicular tumors, breast carcinoma, choriocarcinoma, Kaposi sarcoma, and Letterer-Siwe disorder. VCR, on the other hand, is used primarily to treat hematological malignancies such as acute lymphocytic leukemia, lymphosarcoma, and lymphogranulomatosis, as well as solid tumors in infants [2]. However, the production of these compounds is limited as they are synthesized exclusively in this species and in trace amounts within leaves. Their biosynthesis involves the coupling of two monomeric precursors, vindoline (VDL) and catharanthine (CAT), which are also of pharmacological interest [3, 4]. Both VDL and CAT exhibit antibacterial, antidiabetic, diuretic, and antitumorigenic properties [5–7]. The low yield of VBL and VCR, combined with their high demand, renders the extraction and purification of these compounds both challenging and expensive [2, 8]. The market prices for VBL and VCR are estimated at $5 million/kg and $6.9 million/kg, respectively [9]. The limited production of VBL and VCR, their significant therapeutic potential, along with their high market value, has driven research into alternative production methods beyond traditional plant cultivation. These approaches include *in vitro* plant cell and tissue culture, transgenic plants, and total chemical synthesis, aimed at enhancing production, increasing yield, and meeting growing market demands [10, 11]. However, despite advancements, these methods face critical challenges. *In vitro* cultures and total chemical synthesis suffer from low yields and high costs, while transgenic plants encounter issues with genetic instability, all of which limit their feasibility for industrial-scale production [3, 12, 13].

Since the dimeric alkaloids VBL and VCR are highly cytotoxic to the plant itself, the monomeric units are kept spatially separated in the plant with CAT being secreted onto the leaf surface (in wax exudates) and VDL remaining within the leaf cells (in specialized idioblast/laticifer cells) [4, 14]. The spatial separation of the monomeric precursors provides a biological explanation for the low concentration of the dimeric alkaloids, which are prominently produced only in case of tissue damage. So far, it seems highly unlikely that the *in planta* biosynthetic potential of *C*. *roseus* for DIAs could be enhanced beyond a certain threshold also due to their high cytotoxicity [15]. However, unlike dimeric alkaloids, their monomeric precursors VDL and CAT are abundant in *C*. *roseus* plants [16], hence, one of the best possible means to commercially produce VBL and VCR on an industrial scale is their semi-synthesis from their monomeric precursors extracted from the plant [17].

Since the production in open field may be decreased by climatic conditions, for a continuous supply of these drugs, *C*. *roseus* should be produced stably in quality and quantity [18]. However, *C*. *roseus* is a tropical plant and cannot survive autumn and winter in large parts of the Mediterranean areas, where, in fact, it is marketed as an annual plant; therefore *C*. *roseus* cultivation in controlled environments ensures a stable production of these drugs [19].

Elicitation is one of the most effective techniques for improving the production of secondary metabolites. Elicitors are compounds (abiotic or biotic) that stimulate the plant defense promoting secondary metabolites [20]. Light significantly influences the biosynthesis of VDL and other alkaloids, as well as acidic and basic peroxidase activities [21]. In *C*. *roseus*, red light induces VDL production by increasing the expression of the transcription factor gene GATA1 and VDL pathway genes *T16H2*, *T3O*, *T3R*, *D4H*, and DAT [22]. Similarly, red light treatment (150 μmol m^−2^ s^−1^) considerably enhances the concentration and yield of VDL and CAT in *C*. *roseus* seedlings [23]. Among other plant elicitors, plasma activated water (PAW) is one of the most promising due to its demonstrated efficacy as resistance inducer and to the possibility to be added to LED lights [24–26]. PAW is an aqueous solution characterized by a high concentration of long-lived reactive oxygen and nitrogen species (RONS) which participate in various signalling pathways in plants, regulating the plant development, stress responses, and metabolic processes [27]. RONS trigger a defensive response in the plant tissue consisting in an enhanced production and accumulation of secondary metabolites [28]. Exposure to PAW treatment was reported to enhance the accumulation of bioactive compounds and antioxidant activity of radish sprouts, water spinach and hemp [29–31]. The combined use of light and PAW to increase the alkaloid concentration of *C*. *roseus* of this work aims to maximize the foliar concentrations of VDL and CAT through the cultivation of with artificial light (red LED and white fluorescent lamps) and PAW in indoor farming systems.

## 2. Results

This section exhibits the most important results of this experiment. First of all, chemical analysis of PAW is reported to better characterize this treatment. Second of all, the next section shows the treatments’ effects on plants, describing after ANOVA evaluation of results, VDL and CAT concentrations in leaves and roots.

### 2.1 Chemical analysis of PAW

**Fig 1:**
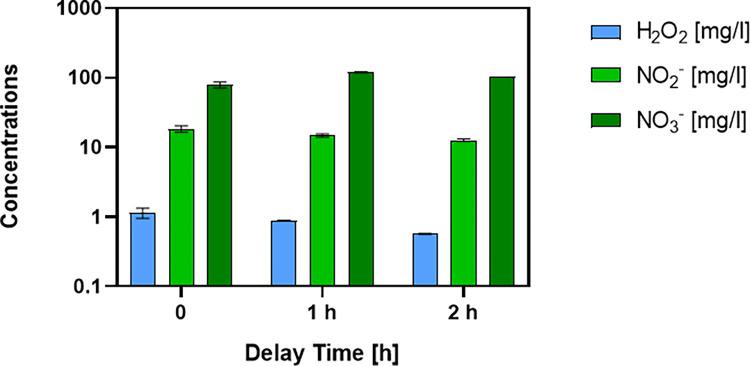
Concentrations of RONS in liquid. In dark green is shown the concentration of nitrates, in light green the concentration of nitrites, and in blue the concentration of peroxides, measured immediately after treatment, 1 hour after treatment, and 2 hours after treatment. Data are presented in logarithmic scale. The bars indicate the standard deviation.

**Fig 2:**
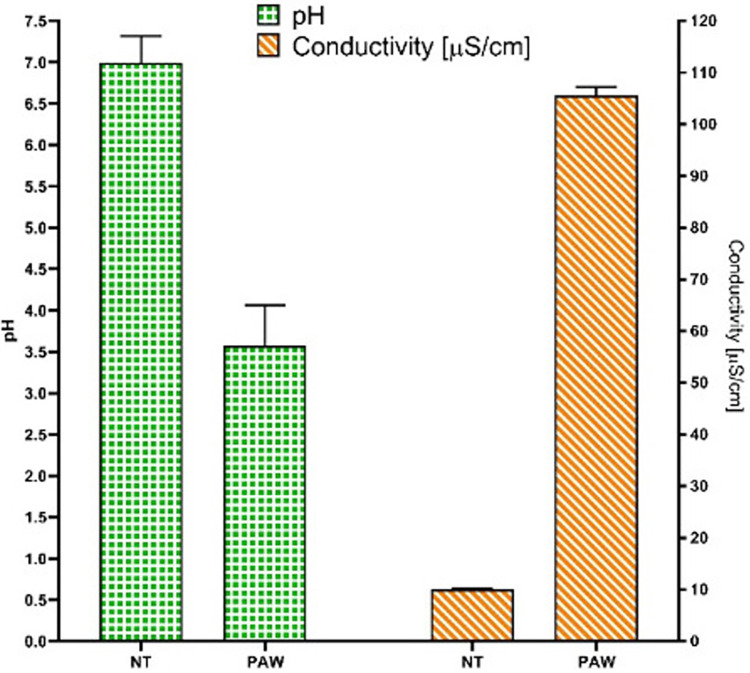
pH and conductivity of non-treated water and PAW. In green is shown the pH, while in orange is shown the conductivity. Both measurements are reported for untreated sterile distilled water (SDW) as well as for PAW. The bars indicate the standard deviation.

S1 Fig in S1 File illustrates voltage and current waveforms pertaining to two consecutive periods, serving as representative instances of the electrical steady-state behavior exhibited by the plasma source during the treatment. The average discharge power, derived from the measured voltage and current, amounts to 114.09 ± 5.56 W. The RONS concentrations contained in PAW after 30 min of plasma treatment were 1.13 ± 0.18 mg/l H_2_O_2_, 18.29 ± 1.94 mg/l NO_2_^-^ and 78.27 ± 7.86 mg/l NO_3_^-^ (Fig 1). As expected, a reduction in the concentrations of H_2_O_2_, NO_2_^-^ was noted reaching a minimum of 0.57 ± 0.003 mg/l and 12.62 ± 0.59 mg/L respectively (Fig 1). Meanwhile, NO3^-^ concentration increases after the plasma treatment, reaching the value of 103.70 ± 0.097 mg/L after 2 h. The pH and conductivity of the liquid at the end of the plasma treatment are 3.57 ± 0.49 and 105.63 ± 1.65 μS/cm, respectively (Fig 2), and resulted stable after 2 h. The increase of the concentration of nitrates and the reduction of the concentration of hydrogen peroxide and nitrites, can be attributed to the well-known process of peroxynitrous acid formation, which occurs from the reaction between hydrogen peroxide and nitrites, leading in part also to the formation of nitrates [32].

### 2.2 Effects of treatments on plants

#### 2.2.1 ANOVA results.

For VDL and CAT leaves concentrations (μg g^-1^ DW), the ANOVA analysis showed a significant difference regarding the main effects of light, PAW and time (p < 0.001 for both VDL and CAT), and for their two-way interactions PAW:time, (p < 0.001 for VDL and p < 0.05 for CAT), light:time (p < 0.001 for VDL and p < 0.05 for CAT), and light:PAW (p < 0.05 for VDL and p < 0.001 for CAT), but not for the light:PAW:time three-way interaction (p = 0.5295 for VDL, p = 0.80134 for CAT). Despite the main effects being statistically significant, since all three two-way interactions were significant, only the means for the light:PAW, light:time, and PAW:time combinations were considered and discussed in the post-hoc analysis, while the means of the main effects were disregarded. This approach was adopted to prevent potential misinterpretations of the results in the presence of significant interactions. However, given the nature of the results, even though the three-way interaction was not significant, it was decided to consider and discuss the means of all Light:PAW:Time combinations.

#### 2.2.2 VDL and CAT concentrations.

**Fig 3:**
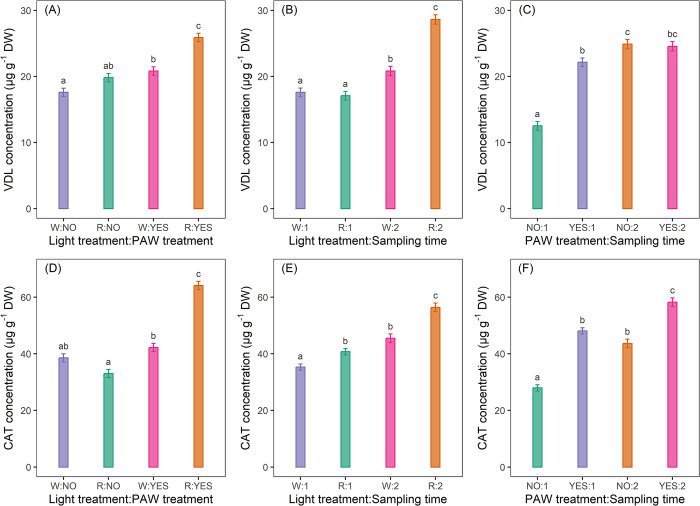
**VDL and CAT concentrations in ***C*. *roseus*** leaves, comparing all combinations of light and plasma activated water (PAW; A and D), light and time (B and E), and PAW and time (C and F). The plants underwent a 61-day pre-treatment acclimatation period, after which they were exposed to four treatments: white light (W, control), red light (R), W+PAW, and R+PAW.** Samples were harvested at 45 (T1) and 70 (T2) days after the end of the pre-treatment period (DAP) to assess temporal variations in VDL and CAT concentrations in response to the experimental treatments. The treatment designations are as follows: 1) for the light and PAW combinations: W:NO represents the treatment with W without PAW (W-T1 and W-T2), R:NO indicates the treatment with R without PAW (R-T1 and R-T2), W:YES denotes the treatment with W and PAW (W+PAW-T1 and W+PAW-T2), and R:YES refers to the treatment with R and PAW (R+PAW-T1 and R+PAW-T2); 2) for the light and time combinations: W:1 includes treatments with W (W and W+PAW) at T1, W:2 comprises treatments with W (W and W+PAW) at T2, R:1 includes treatments with R (R and R+PAW) at T1, and R:2 consists of treatments with R (R and R+PAW) at T2; 3) for the PAW and time combinations: NO:1 encompasses treatments without PAW (W and R) at T1, NO:2 includes treatments without PAW (W and R) at T2, YES:1 represents treatments with PAW (W+PAW and R+PAW) at T1, and YES:2 comprises treatments with PAW (W+PAW and R+PAW) at T2. The bars indicate the standard error (n = 10). Means with different letters within each panel are significantly different at the 5% level by multivariate t distribution (mvt) method for p-value and confidence level adjustment. The highest CAT concentration was in the R:YES treatment (64.1 ± 1.5 μg g^-1^ DW), while the lowest was in the R light treatments without PAW (33.0 ± 1.5 μg g^-1^ DW) (S2 Table in S1 File). R:YES treatment resulted in a significantly higher CAT concentration than all the other treatment combinations: +94.2%, +66.1% and +51.9% compared to R:NO, W:NO and W:YES treatments, respectively. Comparing, however, the light with the same PAW, in the presence of PAW treatment the CAT concentration was significantly higher under R treatment than W treatment (+51.9%), while, without PAW treatment, W-light treatment resulted in a higher CAT concentration than R-light treatment (+17.0%), although there were no statistically significant differences. Then, comparing the PAW with the same light, for the R-grown plants the CAT concentration was significantly higher in the presence of PAW treatment compared to the non-PAW treatment (+94.2%), while for the W-grown plants CAT concentration was higher with PAW treatment than without PAW treatment (+9.3%) although there were not statistically significant differences.

Fig 3A and 3D show VDL and CAT concentrations comparing means of all light:PAW combinations (all against all). The highest VDL concentration was identified in the R:YES treatment (25.9 ± 0.6 μg g^-1^ DW), while the lowest was in the W:NO treatment (17.6 ± 0.6 μg g^-1^ DW) (S1 Table in S1 File). R:YES treatment resulted in a significantly higher VDL concentration than all the other treatment combinations: +47.2%, +30.8% and +24.5% compared to W:NO, R:NO and W:YES treatments, respectively. Comparing, however, the light with the same PAW treatment, in the presence of PAW treatment the VDL concentration was significantly higher in the R-grown plants than in the W-grown plants (+24.5%), while with no PAW treatment the VDL concentration was higher in the R-grown plants than in the W-grown plants (+12.5%), although there were no statistically significant differences. Then, comparing the PAW with the same light, the VDL concentration was significantly higher in the presence of PAW treatment compared to the non-PAW treatment in both R- (+30.8%) and W- (18.2%) grown plants.

The VDL and CAT concentrations comparing means of all light:time combinations (all against all) are shown in Fig 3B and 3E. On a general level the highest VDL concentration was in the R:2 treatments (28.6 ± 0.7 μg g^-1^ DW), while the lowest was in R:1 treatment (17.1 ± 0.6 μg g^-1^ DW) (S1 Table in S1 File). R:2 treatments resulted in a significantly higher VDL concentration compared to all the other treatment combinations: +67.3%, +62.5% and +37.5% compared to R:1, W:1, W:2 treatments, respectively. On the other hand, comparing the light with the same time, at T1 VDL concentration was higher in W treatments than R treatments (+2.9%), although there were not statistically significant differences, while at T2 VDL concentration was significantly higher in treatments with R than in those with W (+37.5%). Furthermore, comparing the time with the same light, the VDL concentration was significantly greater at T2 than at T1 with both R (+67.3%) and W treatments (+18.2%). Concerning CAT, on an overall level the highest CAT concentration was in the R:2 treatments (56.4 ± 1.5 μg g^-1^ DW), while the lowest was in the W:1 treatments (35.3 ± 1.1 μg g^-1^ DW) (S2 Table in S1 File). R:2 treatments resulted in a significantly higher CAT concentration compared to all the other treatments: +59.8%, +38.6% and +24.0% compared to W:1, R:1 and W:2 treatments, respectively. On the other hand, comparing the light with the same time, the CAT concentration was significantly higher in R-light treatments than in those with W-light both at T1 (+15.3%) and at T2 (+24.0%). Furthermore, comparing sampling time with the same light, the CAT concentration was significantly greater in T2 than in T1 both with R (+38.6%) and W (+28.9%) light treatments.

Fig 3C and 3F illustrate VDL and CAT concentrations comparing means of all PAW:time combinations (all against all). On a general level the highest VDL concentration was found in NO:2 treatments (24.9 ± 0.7 μg g^-1^ DW), while the lowest in NO:1 treatment (12.5 ± 0.6 μg g^-1^ DW) (S1 Table in S1 File). NO:2 treatments resulted in a significantly higher VDL concentration compared to NO:1 (+99.2%) and to YES:1 (+12.7%) treatment, but statistically comparable to YES:2 treatments (+1.2% compared to YES:2). The YES:2 treatments, however, produced a significantly higher VDL concentration than the NO:1 treatment (+96.8%). On the other hand, comparing the PAW with the same time, at T1 the VDL concentration was significantly higher (+76.8%) in treatments with PAW compared to treatments without PAW, while at T2 the VDL concentration was greater in treatments without PAW compared to those with PAW (+1.2%) although there were no statistically significant differences. Furthermore, comparing the time with the same PAW, in the absence of PAW treatment the VDL concentration was significantly higher at T2 than at T1 (+99.2%), while in the presence of PAW treatment the VDL concentration was higher at T2 than at T1 (+11.3%) although there were no statistically significant differences. In general, the highest CAT concentration was found in YES:2 treatments (58.2 ± 1.5 μg g^-1^ DW), while the lowest in NO:1 treatment (27.9 ± 1.1 μg g^-1^ DW) (S2 Table in S1 File). YES:2 treatments resulted in a significantly higher CAT concentration than all the other combinations: +108.6%, +33.5% and + 21.0% compared to NO:1, NO:2 and YES:1 treatments, respectively. On the other hand, comparing the PAW with the same time, the CAT concentration was significantly greater in treatments with PAW than in treatments without PAW at both T1 (+72.4%) and T2 (+33.5%). Then, comparing the sampling time with the same PAW treatment, the CAT concentration was significantly higher at T2 than at T1 both with (+21.0%) and without (56.3%) PAW treatments.

Although the three-way interaction Light:PAW:time was not found to be significant in the ANOVA analysis, since the primary objective is to identify the best combination among all treatment combinations to maximize VDL and CAT concentrations (as well as the best combination at each sampling time), it was analyzed the combinations involving all the three experimental factors.

**Fig 4:**
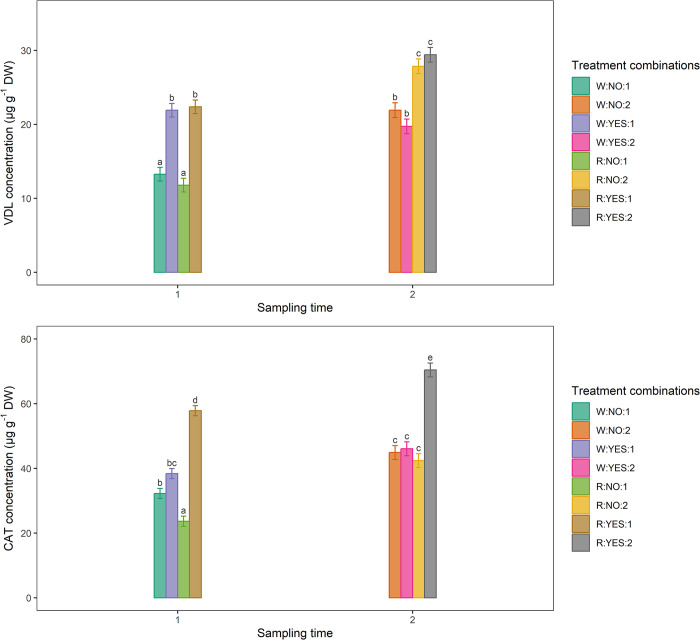
**Concentrations of VDL (A) and CAT (B) in ***C*. *roseus*** leaves under all treatment combinations of light, plasma activated water (PAW), and time. **The plants underwent a 61-day pre-treatment acclimatation period, after which they were exposed to four treatments: white light (W, control), red light (R), W+PAW, and R+PAW. Samples were harvested at 45 (T1) and 70 (T2) days after the end of the pre-treatment period (DAP) to assess temporal variations in VDL and CAT concentrations in response to the experimental treatments. The treatment designations are as follows: W:NO:1 indicates the treatment with W without PAW at T1, W:NO:2 refers to the treatment with W without PAW at T2, W:YES:1 represents the treatment with W and PAW at T1, and W:YES:2 indicates the treatment with W and PAW at T2. Similarly, R:NO:1 designates the treatment with R without PAW at T1, R:NO:2 corresponds to the treatment with R without PAW at T2, R:YES:1 represents the treatment with R and PAW at T1, and R:YES:2 indicates the treatment with R+PAW at T2. The bars indicate the standard error (n = 5). Means with different letters within each panel are significantly different at the 5% level by multivariate t distribution (mvt) method for p-value and confidence level adjustment.

The analysis of all combinations Light:PAW:time (Fig 4) revealed that: (1) regarding VDL (Fig 4A), the highest concentration was identified in R:YES:2 (29.4 μg g-1 DW), while the lowest in R:NO:1 (11.8 μg g-1 DW) (S3 Table in S1 File) and the combination R:YES:2 yielded a significantly higher concentration compared to combinations R:NO:1 (+149.2%), W:NO:1 (+121.1%), W:YES:2 (+49.2%), W:YES:1 (+34.2%), W:NO:2 (+34.2%), and R:YES:1 (+31.3%), but was statistically comparable (though still higher) to R:NO:2 (+5.4%); (2) concerning CAT (Fig 4B), highest concentration was identified in R:YES:2 (70.4 μg g-1 DW), while the lowest in R:NO:1 (23.6 μg g-1 DW) (S3 Table in S1 File) and the R:YES:2 combination resulted in a significantly higher concentration compared to all the other treatment combinations: +198.3%, +118.6%, +83.3%, +66.0%, +56.8%, +52.7% and +21.8% compared to R:NO:1, W:NO:1, W:YES:1, R:NO:2, W:NO:2, W:YES:2, R:YES:1, respectively. Moreover, at individual sampling time level, both T1 and T2 plants treated with R and PAW showed a higher concentration of both alkaloids compared to the other combinations.

#### 2.2.3 VDL and CAT concentrations in roots.

At 45 DAP (T1) one plant was sampled for each treatment to investigate whether, with the experimental treatments used, it had been possible to increase the concentration of these alkaloids in the roots. Therefore, although it was not possible to calculate descriptive statistics such as means and SE (and consequently conduct an ANOVA analysis), these preliminary results (see S2 Fig and S4 Table in the S1 File) are of considerable experimental interest.

## 3. Discussion

### 3.1 Effect of Light:PAW combination on VDL and CAT concentrations

R is considered as a regulator of alkaloid biosynthesis, and its function is dependent on phytochromes and secondary messengers [33, 34]. In *C*. *roseus*, R enhances VDL production by activating the transcription factor GATA1, which regulates light-induced VDL biosynthesis by inducing the promoters of five light-responsive VDL pathway genes [22]. Ohashi-Kaneko et al. [23, 35] reported that irradiating R at a PPFD of 150 μmol m^-2^ s^-1^ using red LEDs was more effective than white FL-based W for increasing the VDL and CAT concentrations, not only in the youngest fully expanded leaves, but also in the total leaves in *C*. *roseus* plant. Fukuyama et al. [36] reported that the yields of VDL and CAT in *C*. *roseus* grown under R tended to be higher than those in plants grown under blue (B), a mixture of R and B (RB; R/ B PPFD ratio was 2/1), and white FL-based W. These results showed that VDL and CAT productions were improved in *C*. *roseus* grown under red light alone.

The effect of plasma activated water (PAW) treatment in crops is mainly regulated by reactive oxygen species (ROS), such as hydrogen peroxide (H_2_O_2_) and reactive nitrogen species (RNS), such as nitrite (NO_2_^-^), and nitrate (NO_3_^-^) [37, 38]. Both ROS and RNS generated by plasma treatment may be easily transported into plant cells and can be a source of nutrients for a plant or act as signaling molecules regulating numerous processes in seeds and plants, including their germination, growth and development [28, 39]. Plasma activated water treatment not only affects plant growth, but it can also regulate the synthesis of secondary metabolites [29, 30]. Indeed, RONS in plants potentially work as elicitors, inducing secondary metabolites by triggering genes involved in the SA and JA synthesis pathway, as well as and pathogenesis related (PR) proteins [40].

Zambon et al. [24] and Contaldo et al. [25] reported that PAW treatment, compared to SDW (sterile distilled water, used to produce the PAW) and FoAl (fosetyl aluminium), in micropropagated periwinkle induced a statistically significant overexpression of *SGD* gene, a key enzyme in the alkaloid’s biosynthetic pathway, involved in the plant defence responses. *SGD* gene plays a pivotal role in the upstream regulation of the pathway leading to the production of CAT, VDL, VCR and VBL. In particular, *SGD* catalyzes the hydrolysis of strictosidine, a critical intermediate in the biosynthetic pathway, leading to the formation of both CAT and VDL through distinct but yet interconnected pathways. The biosynthesis of CAT starts with the hydrolysis of strictosidine by *SGD*, yielding strictosidine aglycone that undergoes enzymatic transformations, first converting into O-acetylstemmadenine, followed by a series of reactions facilitated by specific enzymes, ultimately resulting in CAT. Similarly, VDL synthesis also originates from strictosidine. After hydrolysis by SGD, the strictosidine aglycone serves as a precursor for VDL. It is converted into tabersonine, a key intermediate, which undergoes further enzymatic modifications, leading to the synthesis of VDL [41]. The interconnectedness of these pathways underscores the significance of strictosidine as a common precursor for both CAT and VDL, illustrating SGD’s pivotal role in regulating their biosynthesis.

Zambon et al. [24] and Contaldo et al. [25] attributed SDG activation to the presence of H₂O₂, as supported by Tang et al. [42], who showed that different concentrations of exogenous H_2_O_2_ increased the concentration of alkaloids including VBL, VDL and CAT. Furthermore, recent reports found that nitrate (RNS with the highest concentration in the PAW used) is one of the important nutritional factors affecting alkaloid accumulation in *C*. *roseus* [43]. For instance, in the study by Guo et al. [44] *C*. *roseus* was subjected to UV-B stress and altered levels of nitrate nutrition to investigate their individual and combined effects on growth and alkaloid productions. They concluded that the combination of UV-B radiation and nitrate nitrogen (N- NO_3_^-^) enhanced the accumulation of VDL, CAT and VBL. The enrichment of N nutrition might provide more sources of N for synthesis of alkaloids induced by UV-B light, resulting in increased accumulation of alkaloids. In the present study, PAW also contains nitrites. Therefore, it is assumed that the conclusions drawn by Guo et al. [44] could also extend to NO_2_^-^ which could act as an additional nitrogen source to NO_3_^-^ for the plant. While nitrites can be potentially toxic for most plants, some plants can utilize these RNS as an nitrogen source [45] and the results presented here would suggest that *C*. *roseus* is one of them.

Hence, in accordance with literature [22–25, 35, 36, 42–44] the significantly higher concentrations of VDL and CAT observed with the combination of R and PAW treatment may be attributed to the synergistic effects of R-induced phytochrome activation and PAW-derived RONS. The R likely upregulates key genes in the TIAs biosynthesis pathway, while PAW may enhance this effect by providing signalling molecules (H_2_O_2_, NO_3_^-^, NO_2_^-^) that further stimulate the expression of TIAs enzymes like SGD. Furthermore, it is interesting to note that the combination of W and PAW resulted in higher concentrations of VDL and CAT than sole R. This finding highlights the potential for PAW to enhance the biochemical processes initiated by light treatment. Considering what was reported by Liu et al. [22] and Ohashi-Kaneko et al. [23, 35] regarding the effects of R on the concentrations of VDL (in particular) and CAT, this observation underscores the role of RONS in modulating secondary metabolism, possibly through the activation of both the salicylic acid (SA)/jasmonic acid (JA) pathways and nitrogen assimilation mechanisms, which provide essential precursors for alkaloid biosynthesis.

Furthermore, for both the comparison between PAW at the same light and light at the same PAW, the results agree with what has been reported in literature. The only exception is the comparison of R with W in the absence of PAW treatment for CAT where W treatment resulted in a higher concentration than the one obtained with R-light treatment (although statistically comparable). These results are, therefore, partially in contrast with what was reported by Ohashi-Kaneko et al. [23, 35] and by Fukuyama et al. [36] who did not found significant differences in the concentrations of VDL and CAT among the various treatments, but reported that the content of the two alkaloids tended to be higher under the R treatment.

However, Nagy et al. [46] reported that the concentration of CAT in *C*. *roseus* cultivated under a white multispectral LED treatment with a high percentage of blue ("high blue" treatment) was higher than that in plants grown under the other multispectral LED treatments. Therefore, their results suggest that blue light might play an important role in stimulating CAT production, and this might explain the light higher concentration of CAT under treatment with white fluorescent lamp. In fact, the spectrum emitted by the fluorescent lamp contains a percentage of blue light, while the spectrum emitted by the LED lamp consists only of the red wave band.

### 3.2 Effect of light:time combination on VDL and CAT concentrations

Ohashi-Kaneko et al. [23] investigated the effects of light quality (white -FL- from fluorescent lamps and red -R- from red LEDs) on VDL and CAT concentration and yield of *C*. *roseus* plants sampled at two time points (63 d and 77 d after germination, *i*.*e*., plants grown under LED treatment for 28 d and 42 d, respectively). Overall, the authors reported that the mean concentrations of VDL and CAT were (a) the highest in the R treatment at the second sampling time (77 d after germination); (b) higher at the second sampling time (77 d after germination) than the first (63 d after germination) for both W and R treatment; (c) higher in the R treatment compared to the FL- light treatment at both 63 d and 77 d after germination; (d) higher at the second sampling time compared to the first regardless of the light treatment, revealing an increasing trend of VDL and CAT concentrations with plant age. Furthermore, according to Ohashi et al. [35], at 56 d after germination the VDL and CAT concentrations were higher in the FL-grown plants than in the R-grown plants, whereas the same concentrations were significantly higher in the R-grown plants than in the FL-grown plants after 70 days.

Therefore, the findings presented here appear in agreement with the results of Ohashi et al. [23, 35] for both VDL and CAT and indicate (a) an incremental and non-linear trend of VDL and CAT concentrations over time regardless the type of light treatment; (b) higher effectiveness, at the same sampling time, of R stimulation compared to the W stimulation on CAT and VDL concentrations, starting from a critical time threshold (below which the effect of W remains higher). Despite what was reported by Ohashi et al. [23, 35], at the lower time point (T1 = 45 DAP), the plants grown under W compared to those grown under R, registered a higher (but statistically comparable) concentration for VDL but not for CAT. This difference might be due to the sampling time and consequently to the developmental stage and/or age at which the plant and leaves were at the time of sampling. Indeed, the sampling was carried out not only at two time points but also more distant from each other (45 and 70 DAP vs 56 and 70 after germination and 63 and 77 days after germination in the Ohashi-Kaneko studies), and on plants derived from cuttings (not from seeds) in which the metabolism of these compounds might follow a slightly different temporal trend; (c) maximization effect of VDL and CAT concentrations given by the combination of R and a late time point.

### 3.3 Effect of PAW:time combination on VDL and CAT concentrations

In the study reported by Zambon et al. [24] PAW was used to treat micropropagated periwinkle shoots to evaluate the presence of induced resistance by changes in gene expression analyzed at six time points after PAW treatment: 0 h, 7 h, 24 h, 48 h, 96 h, and 120 h pt (post treatment). These authors–in addition to a statistically significant overexpression of *SDG* gene in PAW-treated plants regardless the sampling time–reported that the SGD level constantly increased over time from 24 to 96 hours, with an overexpression of ca. 5, 5.4 and 6.8 times respectively. The same authors reported that a light overexpression of the *DAT* gene was also registered at the last time point (120 h pt). The *DAT* gene plays an important role in the upstream regulation of the production of VDL [12]. Furthermore, Guo et al. [44] analyzed the VDL and CAT concentrations on ~ 45 d- and ~ 37 d-old plants (the plants were randomized to the different treatments when they were approximately 1 month old and the VDL and CAT analysis were carried out 7d and 15 d after the treatments were applied). These authors found that, regardless the type of treatment, the VDL and CAT concentration increased with plant aging (as reported by Ohashi-Kaneko et al. [23]).

The results presented here confirm what these authors reported; however, irrespective of whether the plants had been treated with PAW or not, the VDL concentration increased over time. Moreover, the PAW treatments at both sampling times did not consistently stimulated a higher VDL concentration compared to the treatments without PAW. Specifically, the YES:2 and YES:1 treatments produced, respectively, lower VDL levels (statistically comparable for the YES:2 treatments and significantly lower for the YES:1 treatments) compared to those obtained with the treatments NO:2.

### 3.4 Effect of Light:PAW:time combination on VDL and CAT concentrations

The higher VDL and CAT concentrations found in the R:YES:2 combination can be explained with what was reported by the authors cited in the previous paragraphs on the combinations Light:PAW, Light:Time and PAW:Time.

## 4. Conclusions

This study investigates, for the first time, the effects of combining red LED light and PAW on the production of VDL and CAT in *Catharanthus roseus* plants. The findings reveal critical insights into optimizing these secondary metabolites, which are essential precursors for anti-cancer drugs. The key conclusions are summarized as follows:

1. *Influence of Plant Age on alkaloids concentrations (optimal harvesting time)*: VDL and CAT concentrations increase with plant age. For growers aiming to maximize the production of these alkaloids, it is advisable to delay harvesting until a later growth stage, *i*.*e*., after a treatment period of 70 days (T2).
2. *Efficacy of PAW*: The use of PAW generally enhances VDL and CAT concentrations compared to treatments without PAW. The effectiveness of PAW tends to increase over time (YES:2 treatments), indicating a cumulative benefit with extended application.
3. *Long-Term PAW Benefits*: Beyond increasing metabolite concentrations, PAW treatment enhance plant biomass and resistance to pathogens [28, 39, 47], contributing to improved crop health and yield. Therefore, for growers aiming to optimize the production of VDL and CAT in *C*. *roseus*, integrating PAW into irrigation systems may represent a promising strategy. The long-term application of PAW may also stimulate various biosynthetic pathways, potentially leading to increased production of other valuable secondary metabolites, such as flavonoids, terpenoids, and phenolic compounds across various plant species. The enhanced accumulation of bioactive compounds may contribute to improved post-harvest conservation and shelf life, thereby ensuring better quality and marketability of the harvested products.
4. *Role of Red Light*: Red light contributes to the accumulation of VDL and CAT, but its effects become significant only when used in conjunction with PAW. Moreover, the cumulative impact of red light (red light with and without PAW) becomes more pronounced with longer treatment durations (R:2 treatments).
5. *Optimal Treatment Combinations*: Regardless the sampling time, the combination of red light and PAW (R:YES treatment) maximizes the concentrations of VDL and CAT. Among all tested combinations, R:YES:2 was found to be the most effective, leading to the highest concentrations of both alkaloids.
6. *Benefits of Treatment Under Harvesting Constraints*: For growers constrained by production strategies that limit the harvest time, applying red light in conjunction with PAW remains advantageous, enhancing the concentrations of VDL and CAT when harvesting cannot be postponed.
7. *Economic Advantages*: The adoption of red light over traditional white light offers economic benefits, primarily due to its energy efficiency. This shift not only supports sustainable cultivation practices but also leads to cost savings.
8. *Resource Allocation Decisions*: In scenarios where PAW treatment is not feasible, utilizing red light alone still provides advantages compared to white light. However, when PAW is available, its integration with red light, particularly at T2, yields optimal returns in terms of VDL and CAT concentrations.
9. *Implications for Agro-Industrial and Pharmaceutical Sectors*: These findings have substantial implications for both the agro-industrial and pharmaceutical industries. Indoor cultivation, utilizing targeted red light and precise PAW applications, can yield higher concentrations of valuable metabolites compared to traditional outdoor methods. Additionally, these systems facilitate reduced water, space, and land usage, optimizing Water Use Efficiency (WUE), Space Use Efficiency (SUE), and Land Use Efficiency (LUE).
10. *Limitations and Future Directions*: While the advantages of indoor cultivation are significant, challenges such as the high initial investment for facility establishment and the varying costs of electricity should be acknowledged. Furthermore, the absence of optimized cultivars for VDL and CAT biosynthesis may limit the effectiveness of these treatments. Future research will focus on conducting varietal trials to identify cultivars with the highest biochemical potential for VDL and CAT production. Additionally, exploring synergistic effects of a higher number of LED spectra in conjunction with PAW technology will be a priority.

In conclusion, this study underscores the critical role of combining red light and PAW in enhancing the production of secondary metabolites, offering valuable strategies for growers to optimize the concentrations of VDL and CAT in *C*. *roseus*.

## 5. Materials and methods

### 5.1 Plant material and growing condition

Unrooted cuttings of *C*. *roseus* were obtained from multiple mother plants maintained for about two weeks (25/01/2022–09/02/2022) in a growth chamber under neutral white tubular fluorescent lamps. On 10/02/2022, shoots of 10–15 cm length were taken from the mother plants, dipped into root hormone powder (Rigenal-P, Cifo) and inserted individually into 1.4 L square plastic pots (D: 10x10 cm; H: 17 cm; Bamaplast) filled with a professional soil mix (SER V-8 14L ANNUALS, Vigorplant). Then, the cuttings were placed in an environmentally controlled growth room (1,78 x 1,78 x 2.35 m) where they were grown for 61 days (10/02/2022–11/04/2022) with 16-h light and 23°C (pretreatment acclimatation period; S3 Fig in S1 File).

Irradiance was provided by parallel neutral white fluorescent tubes (4000 K) and maintained at a photon flux density (PPFD) of ~ 200 μmol m^-2^ s^-1^. PPFD was measured using a LP471/PAR quantum radiometric sensor (Delta Ohm S.r.l., Padua, Italy) at top of the canopy. On 07/04/2022 (56 days after transplant) the plants were tucked with some soil mix. Plants were irrigated as needed with clear tap water from the beginning to the end of the experimental trial. Since *C*. *roseus* is perennial in climate-controlled environment, grows well in a large variety of soils and is known and valued for its durability in dry and nutrient poor situations, no fertilization was given during the trial, to exclude biochemical and metabolic responses due to factors other than the treatments with light and/or PAW. On the 12/04/2022 (61 days after transplant), plants were moved under their respective treatments (described below).

### 5.2 PAW

Plasma-Activated Water (PAW) was generated by subjecting sterile distilled water (SDW) to a plasma source featured as 2 stainless-steel electrodes with a rounded tip, connected to a microsecond pulsed generator (AlmaPulse, AlmaPlasma s.r.l.). The tips of the plasma sources are positioned 0.5 cm above the water’s surface and operate in ambient air. A fixed frequency of 4 kHz is used for plasma generation (S1 Fig in S1 File). A volume of 5 l of SDW, contained in a 10 l plastic (PP) vessel placed on a stirrer (IKA Magnetic Stirrers RCT basic), was directly connected to the ground and exposed to the plasma source. A stirrer speed of 1200 rpm was set. The voltage (V) and the current (i) were measured using a high voltage probe (Tektronix P6015A) and a current probe (Pearson 6585) connected to a digital oscilloscope (Tektronix DPO4034, 350 MHz, 2.5 GSa s^-1^). The average discharge power (P) dissipated over the period (T) was determined by applying the following formula:


$$P=\frac{1}{T}\int_{0}^{T}i(t)*V(t)dt$$ (1)

The concentrations of H_2_O_2_, NO_2_^-^ and NO_3_^-^ induced by plasma in the liquid substrate were measured by means of Amplex® Red Hydrogen Peroxide Assay Kit (Thermo Fisher Scientific, Waltham, MA, USA) and nitrate/nitrite colorimetric assay (ROCHE, Baesel, Switzerland) for 30 min plasma exposure times. Moreover, H_2_O_2_, NO_2_^-^, and NO_3_^-^ were also evaluated 1 h, 2 h after the plasma treatment. Their concentrations were measured according to the manufacturer’s protocols and the absorbances were measured photometrically with a microplate reader (Rayto, P.R. China). The pH measurements were performed with Inolab pH7110 while conductivity measurements with Eutech Instrument COND 6 plus.

### 5.3 Light and PAW treatments

Four treatments (including the control) were applied for 70 days from April 12^th^ until June 20^th^. The treatments used were as follows:

1. White light (W) control (CK), from parallel neutral fluorescent tubes (FL, 4000 K), PPFD ~ 150 μmol m^-2^ s^-1^
2. Red light (R) from top-light LED lamp (C-LED Top-lighting 150 W, 660 nm peak wavelenght), PPFD ~ 150 μmol m^-2^ s^-1^
3. W+ PAW, PPFD ~ 150 μmol m^-2^ s^-1^
4. R + PAW, PPFD ~ 150 μmol m^-2^ s^-1^

These treatments were tested in two sampling times (“T1” and “T2”), to assess the temporal variation in VDL and CAT concentrations in response to the different treatment conditions. The plants were exposed to light treatments in the growth room over the course of the whole experiment.

The photoperiod and the temperature of the chamber were the same as in the pretreatment acclimatation period (16/8 h and 23°C, respectively). Light intensity (PPFD) at canopy heigh was maintained constant at ~ 150 μmol m^-2^ s^-1^. Canopy-level PPFD was measured with the quantum radiometric sensor used in the pretreatment acclimatation period (LP471/PAR, Delta Ohm S.r.l., Padua, Italy). Red monochromatic light was selected as experimental treatment because, as mentioned in the introduction, previous studies by Liu et al. [22], Fukuyama et al. [36] and Ohashi-Kaneko et al. [23, 35] found that this wavelength exerts a particularly stimulating effect on the production of VDL and CAT. The choice of PPFD at 150 μmol m^-2^ s^-1^ followed the same rationale, as Fukuyama et al. [18] identified this intensity as optimal for maximizing VDL and CAT concentrations. Similarly, PAW, which is ideally suited for its combined use with LEDs in controlled environments, was chosen for its general stimulating effects on plant secondary metabolism [28–31] as mentioned in the introduction, and for its role in the overexpression of genes involved in TIA biosynthesis,.

Two PAW treatments were performed: on April 12^th^ (61 days after transplant) and May 19^th^ (98 days after transplant). On April 12, 2022, before being subjected to light quality treatments (red LED lamps and white fluorescent tubes, PPFD ~ 150 μmol m^-2^ s^-1^), the plants belonging to the different experimental treatments were taken from the growth chamber and transferred to a separate facility where the treatment was applied.

**Fig 5:**
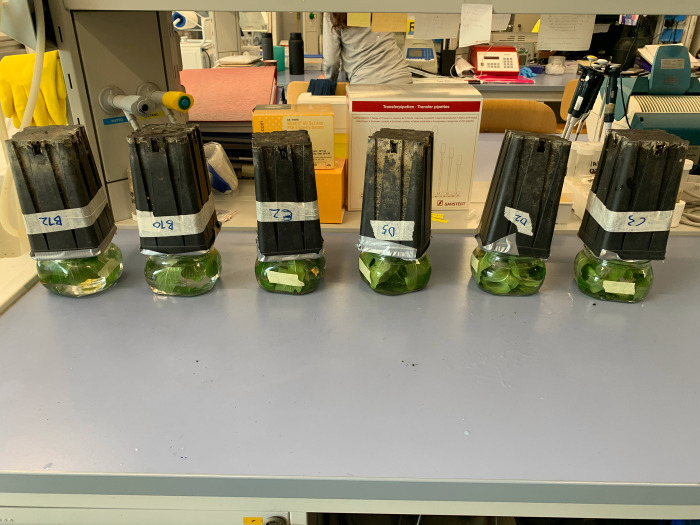
Treatment with PAW by upside down sub-immersion method. Plants belonging to W+PAW and R+PAW treatments were treated for 20 minutes by immersing the aerial apparatus; plants belonging to W and R treatments were treated for 20 minutes with SDW from which the PAW was obtained (control) using the same method.

**Fig 6:**
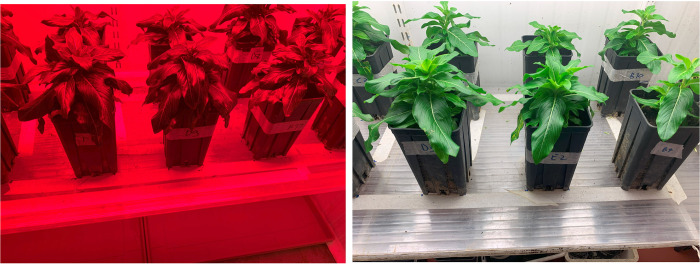
Plants subjected to experimental treatments. After immersing in PAW and SDW, the plants were placed under the two light sources: red LED (left) and white fluorescent lamps (right).

Before being transferred, plants chosen for PAW treatment were randomly selected. The periwinkle plants of the W+PAW and R+PAW treatments were immersed upside down for 20 min in jars containing 0,5 L of PAW, while the plants of the W and R treatments were immersed, as a control, upside down for 20 min in 0,5 L of SDW from which PAW was produced (Fig 5). The aerial apparatus of the plant was isolate from the ground preventing the latter to be in contact with the PAW and SDW once the plant was overturned (S4 Fig in S1 File). PAW treatment was completed within one hour of PAW production. After the immersion in PAW and SDW, the plants were randomly allocated to their respective light treatments (Fig 6). The same procedure was applied for the May 19^th^ PAW treatment.

### 5.4 Sampling for alkaloids analysis

To determine alkaloids concentration (μg g^-1^ DW) samplings were carried out at T1 (26/05/2022, 45 days after the end of pretreatment -DAP-, 106-d after the transplanting of the cuttings) and T2 (20/06/2022, *70* DAP, 131-d after the transplanting of the cuttings) on the same plants. At T1, one plant was randomly harvested for each experimental treatment to analyze VDL and CAT concentration in roots. From the remaining five plants of each treatment, three leaves were randomly removed in three different portions of the canopy (basal, middle and upper) and used for the measurement of leaf VDL and CAT concentrations. The same procedure was repeated at T2, using the plants sampled at T1 from each experimental treatment. The selection of sampling times T1 (45 DAP) and T2 (70 DAP) was influenced by several factors, including the need to adapt the protocol to the characteristics of the plants obtained from cuttings rather than seeds, which were instead in the studies by Ohashi-Kaneko et al. [23, 35] and Fukuyama et al. [18, 36]. In the present experimental conditions, the cuttings required a longer period to recover from transplant stress and to reach optimal biomass before being subjected to treatments with red LED light and PAW. Consequently, the pre-treatment under white light lasted 61 days, compared to the 35 days used by Ohashi-Kaneko et al. [23, 35] and Fukuyama et al. [18, 36] in their studies on germinated seedlings. Ohashi-Kaneko et al. conducted two studies [23, 35] in which plants were sampled at different time points following a white light pre-treatment: in the first study [35], plants were sampled at 56 and 70 days after germination, while in the second study [23], sampling occurred at 63 and 77 days after germination. Although their experiments started from seeds, the sampling times provide an important reference for monitoring the dynamics of metabolite accumulation. In particular, that starting from cuttings might result in slower plant development and that the plants might requir more time to recover from transplant stress. Therefore, it was advantageous to not only sample at more advanced time points compared to those used by Ohashi-Kaneko et al. [23, 35] but also to increase the temporal distance between the two sampling events. This allows to better observe the evolution of VDL and CAT concentrations at later developmental stages. In summary, the sampling at 45 (T1) and 70 (T2) DAP was chosen not only to reference previous studies but also to adapt the experimental approach to the physiological needs of the cuttings and to investigate the variation in concentrations of VDL and CAT at more advanced and temporally spaced time points compared to studies conducted on plants germinated from seeds.

### 5.5 Quantitative analysis of alkaloids

LC-DAD-MS/MS analysis was performed on a Waters Alliance e2695 chromatographic system with autosampler coupled to a Waters 2998 photo diode array detector and a Waters Micromass Quattro Micro triple-quadrupole mass spectrometer equipped with an electrospray ion source working in positive ionisation mode (ESI+). Data processing was performed using Waters MassLynx 4.1 software. Separations were obtained on a Waters XTerra MS C18 column (100 × 2.1 mm I.D., 3.5 μm), maintained at room temperature and equipped with a Waters VanGuard XTerra C18 guard column. The mobile phase was a mixture of 0.15% aqueous formic acid (component A) and 0.15% formic acid acetonitrile (component B), flowing at a constant rate of 0.3 mL/min. The gradient program of the mobile phase started with A:B 90:10 (V/V) maintained for 1 min, then ramped to A:B 70:30 (V/V) over 1 min; this ratio was maintained for 3 min, then ramped to A:B 50:50 (V/V) over 1 min and maintained for 3 min, then ramped to the initial conditions over 1 min and was maintained at this ratio for 1 min to allow column re-equilibration. The two components of the mobile phase were filtered through Sartorius (Göttingen, Germany) membrane filters (47 mm diameter, polyamide, 0.2 μm pore size) and degassed by an ultrasonic bath. For LC-DAD-MS/MS quantitative analysis of VDL and CAT DAD detector scanned from 220 to 400 nm, while multiple reaction monitoring (MRM) transitions were used for MS detection, acquiring in ESI+ mode. All the MS parameters were optimised for maximum abundance of the interested ions via direct infusion of reference standard solution of the target analyte (1 μg/mL methanolic solutions) at 20 μL/min. The optimised parameters were as follows: ion source voltage, 3.5 kV; ion source temperature, 125°C; desolvation temperature, 380°C; desolvation gas flow, 600 L/h; extractor potential, 3.2 V; collision exit potential, 1 V. N_2_ was used as the desolvation gas, while Ar was used as the collision gas. The precursor ion and the product ion, with dwell time, cone voltage and collision energy, were optimised for each analyte (Table 1).

**Table 1:** Analyte-dependent MRM MS parameters.

Compound	MW (g/mol)	Parent ion (*m/z*)	Product ions (*m/z*)^a^	Cone voltage (V)	Collision energy (eV)
CAT	336.4	337.5	173.4, *144*.*3*	25	30
VDL	456.6	457.5	427.5, *397*.*3*	35	40
IS (VDL-d3)	459.6	460.5	430.4, *400*.*2*	35	40

^a^ In italic, qualifier ions

Samples were dried in a stove at 40°C until constant weight, grounded in an IKA (Staufen, Germany) A11-1 electric analytical mill and then 0.25 g of powdered material were extracted with 2.5 mL of 70% (V/V) ethanol for 24 h under continuous stirring. The procedure was repeated three times, then the pooled extract was filtered on a Büchner funnel, and the solvent was evaporated in a vacuum concentrator (Savant SpeedVac SPD210, Thermo Fisher Scientific, Waltham, MA, USA) at 40°C to yield the crude extracts. Suitable amounts of extracts were dissolved in methanol to obtain working solutions at the concentration of 1 mg/mL. The obtained solutions were then centrifuged, the supernatant was filtered on 25 mm Ø, 0.45 μm pore size nylon syringe filters, then appropriately diluted and subjected to microextraction by packed sorbent (MEPS) clean-up procedure. MEPS procedure was performed on a SGE Analytical Science (Melbourne, VIC, Australia) apparatus, consisting of a 100 μL HPLC syringe with a removable needle, fitted with a BIN (barrel insert and needle) containing an M1 (C8+SCX mixed mode) sorbent. The sorbent was activated with 200 μL of methanol and then equilibrated with 200 μL of ultrapure water. The loading solution was a mixture of 100 μL of diluted methanolic extracts, 100 μL of ultrapure water and 5 μL of a solution containing vindoline-d3 (used as internal standard–IS); the loading mixture was drawn into the syringe and discharged back 10 times. The sorbent was washed with 100 μL of water, 100 μL of a water/methanol mixture (90:10, V:V) and then eluted by drawing and discharging 500 μL of methanol. The eluate was dried under vacuum, redissolved with 100 μL of a component A:component B (70:30, V/V) mixture and injected into the LC-DAD-MS/MS system. The analytical method used in this study demonstrated a Limit of Detection (LOD) of 0.1 μg g^-1^ DW for VDL and 0.2 μg g^-1^ DW for CAT. Additionally, the Limit of Quantification (LOQ) was established at 0.3 μg g^-1^ DW for VDL and 0.6 μg g^-1^ DW for CAT.

### 5.6 Statistical analysis

All statistical analyses were executed in RStudio version 4.2.2 [48]. Since the samples were always collected from the same plants at the different sampling times (T1 and T2), a repeated measures design was used to investigate the effects of light treatments, PAW treatments, sampling time and their interactions on VDL and CAT concentrations. The experimental design comprised four distinct treatments, with each treatment featuring five individual plants as biological replicates and 10 individual plants as technical replicates. To analyze the data a linear mixed-effects model (LME) was employed. The model was fitted using the “lme” function form the “nlme” package [49] and is the same for both VDL and CAT as follows:

*lme (Concentration ~ light + PAW + time + light:time + PAW:time + light:PAW + light:PAW:time, random = ~ time|plant, data = dataset)*

Where:

- the response variable is represented by *Concentration*
- the fixed-effects structure of the model is given by the main effects of *light* (“Light treatment”, two levels: “W”, “R”), *PAW* (“PAW treatment”, two levels: “YES”, “NO”), *time* (“Sampling time (T)”, two levels: “1”, “2”) factors and by their interactions. This comprehensive fixed-effects structure allowed us to explore potential interactions between the variables of interest.
- the random-effects structure of the model is represented by the effects of *time* (random slope) and *plant* factors (“Plants used in the experiment on which repeated measurements were taken”; random intercept). The selection of a linear mixed-effects model with random intercept and slope was motivated by the repeated measures design of the experiment. Indeed, this model accounts for the correlation between observations from the same plant over time (random slope term, that captures variability in the rate of change in concentration over time within each plant), acknowledging that individual plants may exhibit unique baseline concentration levels and variable responses over time (random intercept term, that accounts for variability in baseline concentration among different plants).

The P-values for fixed effects were obtained using the “*Anova*” (type III) function of the “*car*” package [50]. Post-hoc pairwise comparisons of the means were conducted using the multivariate t distribution (mvt) method for P-value and confidence-level adjustment, using the functions “*emmeans*” and “*compact letter display (cld)*” from the “*emmeans*” and “*multcomp*” packages, respectively [51, 52]. The mvt adjustment was chosen for the post-hoc analysis due to the nature of the repeated measures experimental design, in which the observations are not independent but are correlated over time. Repeated measures on the same subjects can lead to correlations among the observations, making the mvt approach particularly useful as it accounts for this dependency, a fundamental characteristic of repeated measures designs. The mixed model employed captures this complexity through random effects, while the mvt adjustment effectively manages the correlations, unlike other tests that assume independence. Additionally, the mvt method aligns seamlessly with the estimated marginal means (emmeans) derived from the mixed model, facilitating accurate correction of multiple comparisons while precisely respecting the familywise error rate (FWER) in a context of complex variance. This choice ensures more robust and reliable estimates compared to simpler methods, thereby contributing to the integrity of the analysis results.

The significance level was set to α = 0.05. The assumptions of normality and homogeneity of variance were confirmed by a quantile-quantile plot and a residual plot respectively. Data visualization was performed using the "*ggplot*" function in the “*ggplot2*” package, version 3.4.1 [53]. Results were expressed as mean ± SE (standard error), excepting for the S3 Table in S1 File relate to roots where the total treatments VDL and CAT concentrations are expressed only as means and for the chemical analysis of PAW where they are expresses as mean ± SD (standard deviation).

## Supporting information

S1 File (DOCX)

## Abbreviations:

T16H2: Tabersonine-16-hydroxylase2
T3O: Tabersonine-3-oxygenase
T3R: Tabersonine-3-reductase
D4H: Desacetoxyvindoline-4-hydroxylase
DAT: Deacetylvindoline O-acetyltransferase
SDG: Strictosidine-β-glucosidase.
