# Supplementary material for: Enhancement of vindoline and catharanthine production in Catharanthus roseus by LED light and plasma activated water
Source: PLoS One. 2024 Dec 31;19(12):e0315542. doi: 10.1371/journal.pone.0315542 (PMC12527298; doi:10.1371/journal.pone.0315542)
Supplement: S1 File — (DOCX) [file pone.0315542.s001.docx]

**Supplementary material**

| 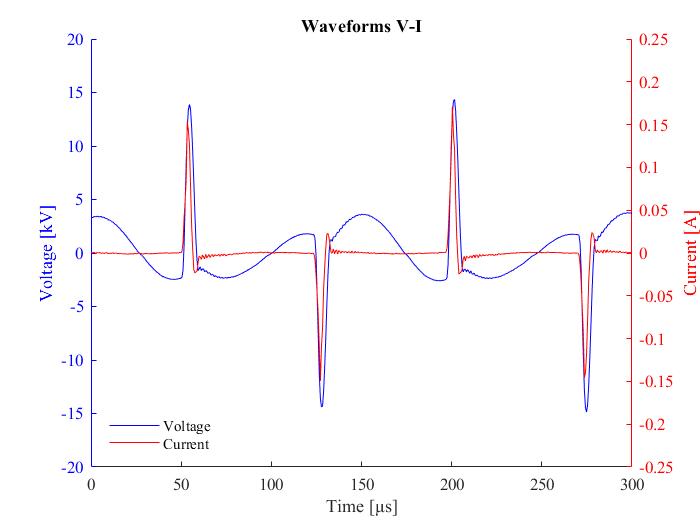 |
| --- |
| **Fig S1**. **Electrical measurements of the plasma discharge**. In blue is reported the temporal evolution of the voltage [kV] and in red is reported the temporal evolution of the current [A] during the treatment. |

| **Table S1. VDL concentration (µg g-^1^ DW) in *C.roseus* leaves** | | | | | | | | |  |
| --- | --- | --- | --- | --- | --- | --- | --- | --- | --- |
|  |  |  |  |  |  |  |  |  |  |
|  |  |  |  |  |  |  |  |  |  |
| *PAW:time* | | | *Light:time* | | | *Light:PAW* | | |  |
|  |  |  |  |  |  |  |  |  |  |
| NO:1 | 12.5 ± 0.6 a | | W:1 | 17.6 ± 0.6 a | | W:NO | 17.6 ± 0.6 a | |  |
|  |  |  |  |  |  |  |  |  |  |
| NO:2 | 24.9 ± 0.7 c | | W:2 | 20.8 ± 0.7 b | | W:YES | 20.8 ± 0.6 b | |  |
|  |  |  |  |  |  |  |  |  |  |
| YES:1 | 22.1 ± 0.6 b | | R:1 | 17.1 ± 0.6 a | | R:NO | 19.8 ± 0.6 ab | |  |
|  |  |  |  |  |  |  |  |  |  |
| YES:2 | 24.6 ± 0.7 bc | | R:2 | 28.6 ± 0.7 c | | R:YES | 25.9 ± 0.6 c | |  |
|  |  |  |  |  |  |  |  |  |  |
| Values are means ± standard error (n = 10). Means with different letters are significantly different at the 5% level by a pairwise comparison approach based on the multivariate t distribution (mvt) method for p-value and confidence level adjustment. | | | | | | | | |  |
|  |  |  |  |  |  |  |  |  |  |
|  |  |  |  |  |  |  |  |  |  |

| **Table S2. CAT concentration (µg g-^1^ DW) in *C.roseus* leaves** | | | | | | | | |  |
| --- | --- | --- | --- | --- | --- | --- | --- | --- | --- |
|  |  |  |  |  |  |  |  |  |  |
|  |  |  |  |  |  |  |  |  |  |
| *PAW:time* | | | *Light:time* | | | *Light:PAW* | | |  |
|  |  |  |  |  |  |  |  |  |  |
| NO:1 | 27.9 ± 1.1 a | | W:1 | 35.3 ± 1.1 a | | W:NO | 38.6 ± 1.5 ab | |  |
|  |  |  |  |  |  |  |  |  |  |
| NO:2 | 43.6 ± 1.5 b | | W:2 | 45.5 ± 1.5 b | | W:YES | 42.2 ± 1.5 b | |  |
|  |  |  |  |  |  |  |  |  |  |
| YES:1 | 48.1 ± 1.1 b | | R:1 | 40.7 ± 1.1 b | | R:NO | 33.0 ± 1.5 a | |  |
|  |  |  |  |  |  |  |  |  |  |
| YES:2 | 58.2 ± 1.5 c | | R:2 | 56.4 ± 1.5 c | | R:YES | 64.1 ± 1.5 c | |  |
|  |  |  |  |  |  |  |  |  |  |
| Values are means ± standard error (n = 10). Means with different letters are significantly different at the 5% level by a pairwise comparison approach based on the multivariate t distribution (mvt) method for p-value and confidence level adjustment. | | | | | | | | |  |
|  |  |  |  |  |  |  |  |  |  |
|  |  |  |  |  |  |  |  |  |  |

| **Table S3. VDL and CAT concentrations (µg g-^1^ DW) in *C.roseus* leaves under all combinations** | | |  |
| --- | --- | --- | --- |
|  |  |  |  |
|  |  |  |  |
|  | *VDL* | *CAT* |  |
|  |  |  |  |
| W:NO:1 | 13.3 ± 0.918 | 32.2 ± 1.57 |  |
|  |  |  |  |
| W:NO:2 | 21.9 ± 0.996 | 44.9 ± 2.14 |  |
|  |  |  |  |
| W:YES:1 | 21.9 ± 0.918 | 38.4 ± 1.57 |  |
|  |  |  |  |
| W:YES:2 | 19.7 ± 0.996 | 46.1 ± 2.14 |  |
|  |  |  |  |
| R:NO:1 | 11.8 ± 0.918 | 23.6 ± 1.57 |  |
|  |  |  |  |
| R:NO:2 | 27.9 ± 0.996 | 42.4 ± 2.14 |  |
|  |  |  |  |
| R:YES:1 | 22.4 ± 0.918 | 57.8 ± 1.57 |  |
|  |  |  |  |
| R:YES:2 | 29.4 ± 0.996 | 70.4 ± 2.14 |  |
|  |  |  |  |
| Values are means ± standard error (n = 5). Means with different letters are significantly different at the 5% level by a pairwise comparison approach based on the multivariate t distribution (mvt) method for p-value and confidence level adjustment. | | |  |
|  |  |  |  |
|  |  |  |  |
|  |  |  |  |

*VDL and CAT concentrations in roots*

Regarding VDL unlike what is reported in the literature (i.e. unlike CAT which is produced both in the aerial and underground organs of the plant, VDL is synthesized only in the green aerial parts of the plant and not in the roots; [1,2])our results show how vindoline is also produced in the roots of the plant. It seems that in roots (Fig. S2A and Table S3) at T1 the R:YES treatment determined a considerably higher concentration than all the other treatments (+260.6%, + 249.7% and +140.4%, compared to the R:NO, W:YES and W:NO treatments, respectively) and also to the same treatment in leaves (+157.6%). Furthermore, contrary to what was observed in the leaves, the W:NO treatment resulted in a higher concentration than the W:YES treatment (+51.5%) and the R light treatment seem to have had a significantly higher effect than the light treatment W (+82.0%; in leaves -2.8% compared to W light). Similarly to what was observed in the leaves, the treatment with PAW seems to have determined a higher concentration than the treatment without PAW (+82.0%). Finally, the total mean of the treatments at T1 resulted considerably higher in the roots than in the leaves (+64.4%) suggesting a higher mean total level of VDL in the hypogeal apparatus. Concerning CAT, in roots (Fig. S 2B and Table S3) at T1 the R:NO treatment determined a higher concentration than all the other treatments (+78.0%, +38.3% and +5.5%, compared to the R:YES, W:YES and W:NO, respectively) and also to the same treatment in the leaves (+13.1%). Furthermore, contrary to what was observed in the leaves, the W:NO treatment determined a higher concentration than the W:YES treatment (+31.1%), the W light treatment had a greater effect than the W light treatment ( +7.0%; in the leaves, on the other hand, -13.3% compared to W light) and the treatment without PAW have had a greater effect than the treatment with PAW (+48.7%). Finally, the total mean of treatments at T1 would appear to be higher in the leaves than in the roots (+75.9%) suggesting a higher mean total level of VDL in the aerial system than in the roots. Larger sampling size will be used to confirm these results, as well as one or more sampling times to verify, likewise for the leaves, the variation of the concentrations of these two alkaloids as a function of time.

| 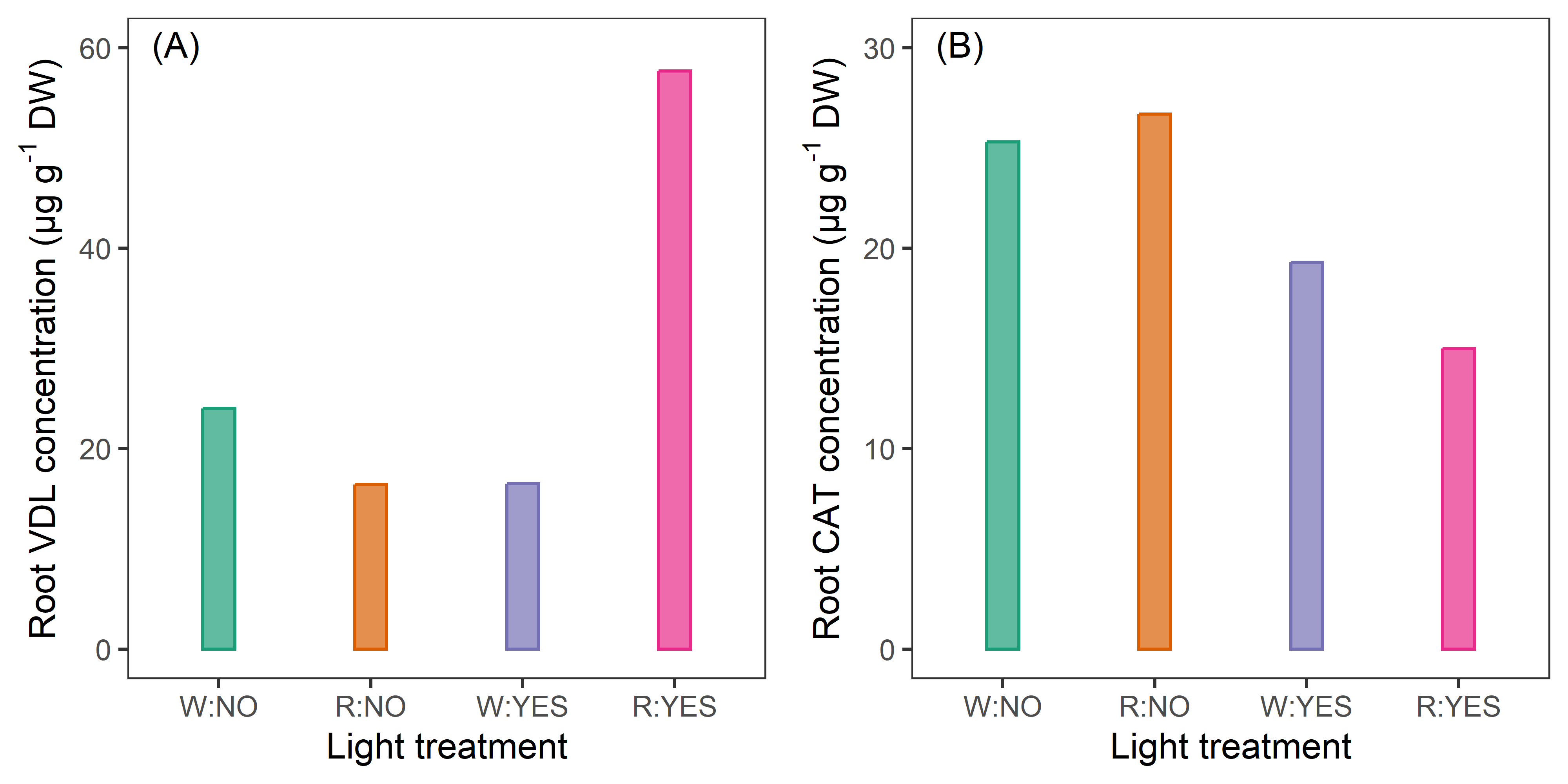 |
| --- |
| **Fig S2**. **VDL (A) and CAT (B) concentrations in *C. roseus* roots at T1, 45 days after the end of pretreatment (DAP) .** W:NO represents the treatment with W without PAW (W-T1 and W-T2), R:NO indicates the treatment with R without PAW (R-T1 and R-T2), W:YES denotes the treatment with W and PAW (W+PAW-T1 and W+PAW-T2), and R:YES refers to the treatment with R and PAW (R+PAW-T1 and R+PAW-T2).The plants were grown under four treatments: white light (W, control), red light (R), W + plasma-activated water (PAW), and R + PAW. At 45 DAP (T1), one plant per treatment was sampled to analyse and compare differences in VDL and CAT concentration in roots. |

| **Table S4. VDL and CAT concentrations in *C.roseus* leaves and roots at 45 DAP (T1)** | | | | |  |
| --- | --- | --- | --- | --- | --- |
|  |  |  |  |  |  |
| Treatments | VDL concentration (µg g^-1^ DW) | | CAT concentration (µg g^-1^ DW) | |  |
|  |  |  |  |  |  |
|  |  |  |  |  |  |
|  | *Leaves* | *Roots* | Leaves | Roots |  |
|  |  |  |  |  |  |
| W: NO | 13.3 | 24.0 | 32.2 | 25.3 |  |
|  |  |  |  |  |  |
| R:NO | 11.8 | 16.0 | 23.6 | 26.7 |  |
|  |  |  |  |  |  |
| W:YES | 21.9 | 16.5 | 38.4 | 19.3 |  |
|  |  |  |  |  |  |
| R:YES | 22.4 | 57.7 | 57.8 | 15.0 |  |
|  |  |  |  |  |  |
| *Total mean* | *17.4* | *28.6* | 38.0 | 21.6 |  |
|  |  |  |  |  |  |
| Values are means and single measurements in leaves and roots, respectively. | | | | |  |
|  |  |  |  |  |  |
|  |  |  |  |  |  |

| 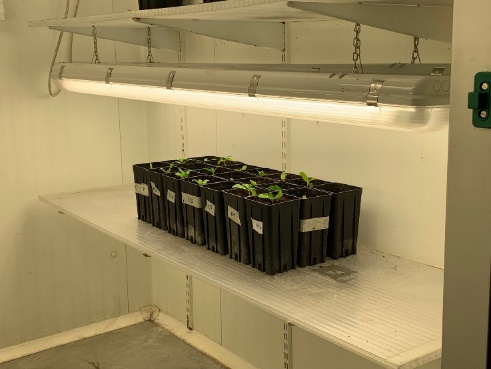 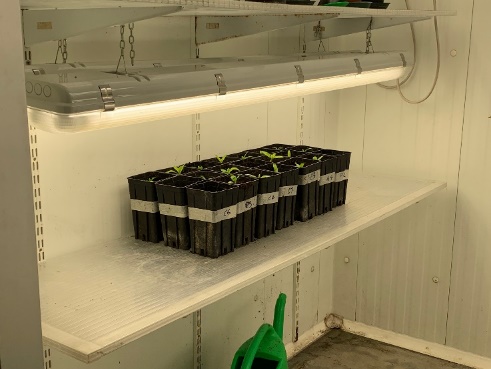 |
| --- |
| **Fig S3. Plants during the pretreatment acclimatation period.** Before exposure to experimental treatments, plants were grown for 61 days in an environmentally controlled room with a 16-hour light period, a temperature of 23ºC, and a PPFD of ~ 200 μmol m⁻²s⁻¹ provided by white fluorescent lamps. |

| *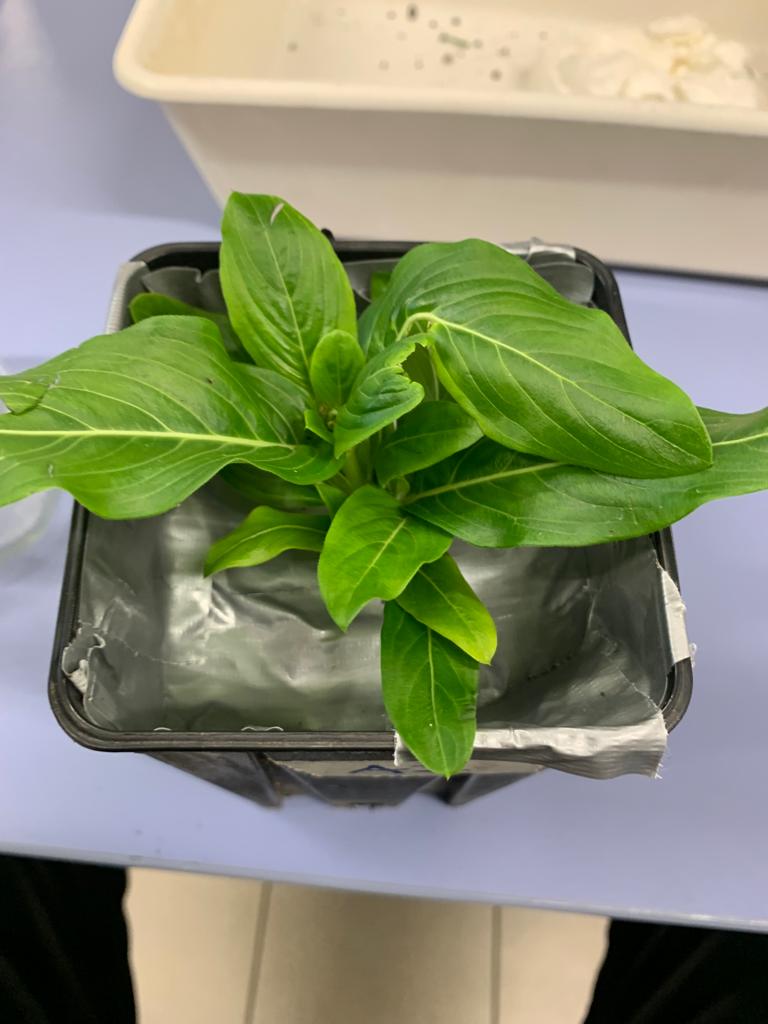 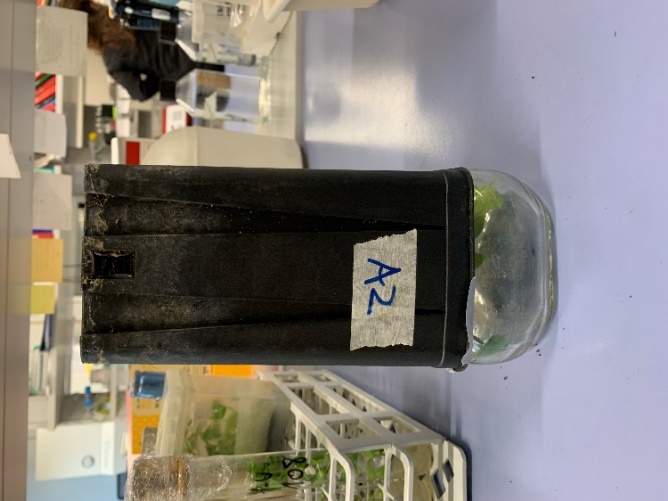* |
| --- |
| **Fig S4.** **Preparation of plants for PAW treatment for upside down sub-immersion.** The aerial part of the plant was inserted into the jar by turning the vase upside down and inserting the internal square base inside the jar, so that it rests on the circular perimeter of the latter. American adhesive tape for plumbing was used to isolate the aerial apparatus of the plant was isolate from the ground, preventing the latter from coming into contact with the PAW and SDW once the plant was overturned. |

*References*

[1] Mall M, Shanker K, Samad A, Kalra A, Sundaresan V, Shukla AK. Stress responsiveness of vindoline accumulation in Catharanthus roseus leaves is mediated through co-expression of allene oxide cyclase with pathway genes. Protoplasma 2022;259:755–73. https://doi.org/10.1007/S00709-021-01701-6.

[2] Shukla A, Khanuja S. Catharanthus roseus: The metabolome that represents a unique reservoir of medicinally important alkaloids under precise genomic regulation. OMICS Applications in Crop Science (Ed D Barh) 2013:325–84.
